# Correction for Kibler et al., “Replication-Competent NYVAC-KC Yields Improved Immunogenicity to HIV-1 Antigens in Rhesus Macaques Compared to Nonreplicating NYVAC”

**DOI:** 10.1128/JVI.00968-19

**Published:** 2019-10-15

**Authors:** Karen V. Kibler, Benedikt Asbach, Beatriz Perdiguero, Juan García-Arriaza, Nicole L. Yates, Robert Parks, Sherry Stanfield-Oakley, Guido Ferrari, David C. Montefiori, Georgia D. Tomaras, Mario Roederer, Kathryn E. Foulds, Donald N. Forthal, Michael S. Seaman, Steve Self, Raphael Gottardo, Sanjay Phogat, James Tartaglia, Susan Barnett, Anthony D. Cristillo, Deborah Weiss, Lindsey Galmin, Song Ding, Jonathan L. Heeney, Mariano Esteban, Ralf Wagner, Giuseppe Pantaleo, Bertram L. Jacobs

**Affiliations:** a Biodesign Institute, Arizona State University, Tempe, Arizona, USA; b Institute of Medical Microbiology and Hygiene, University of Regensburg, Regensburg, Germany; c Department of Molecular and Cellular Biology, Centro Nacional de Biotecnología, Consejo Superior de Investigaciones Científicas, Madrid, Spain; d Duke University Medical Center, Durham, North Carolina, USA; e Vaccine Research Center, National Institute of Allergy and Infectious Diseases, National Institutes of Health, Bethesda, Maryland, USA; f Division of Infectious Diseases Department of Medicine, Irvine School of Medicine, University of California, Irvine, California, USA; g Center for Virology and Vaccine Research, Beth Israel Deaconess Medical Center, Boston, Massachusetts, USA; h Statistical Center for HIV/AIDS Research and Prevention, Fred Hutchinson Cancer Research Center, Seattle, Washington, USA; i Sanofi Pasteur, Swiftwater, Pennsylvania, USA; j Novartis Vaccines and Diagnostics, Inc., Cambridge, Massachusetts, USA; k Advanced BioScience Laboratories, Inc., Rockville, Maryland, USA; l EuroVacc Foundation, Lausanne, Switzerland; m Lab of Viral Zoonotics, Department of Veterinary Medicine, University of Cambridge, Cambridge, United Kingdom; n Institute of Clinical Microbiology and Hygiene, University of Regensburg, Regensburg, Germany; o Division of Immunology and Allergy, Department of Medicine, Centre Hospitalier Universitaire Vaudois, University of Lausanne, Lausanne, Switzerland; p School of Life Sciences, Arizona State University, Tempe, Arizona, USA

## AUTHOR CORRECTION

Volume 93, no. 3, e01513-18, 2019, https://doi.org/10.1128/JVI.01513-18. The article was originally published on 17 January 2019 with a standard copyright line (“© 2019 American Society for Microbiology. All Rights Reserved.”). We elected to pay for open access for the article after publication, necessitating replacement of the original copyright line with “© 2019 Kibler et al. This is an open-access article distributed under the terms of the Creative Commons Attribution 4.0 International license.” This change was made to the online version of the article on 21 August 2019.

